# Curricula Innovation to Positively Influence Preference for Working With Older Adults: A Review of the Literature

**DOI:** 10.1093/geroni/igaa057.1780

**Published:** 2020-12-16

**Authors:** Christine Brewer

**Affiliations:** Villanova University, Villanova, Pennsylvania, United States

## Abstract

Few nursing students show preference in working with older adults. The purpose of this study was to review the U.S. nursing education evidence-based literature to determine curricula innovation to positively influence preference for working with older adults. CINAHL, Medline, Ovid Emcare, PsychInfo, and PubMed databases were searched for relevant U.S studies published between 2009 and 2020 using the search terms “nursing students”, “geriatrics OR gerontology OR older adults OR elderly OR aging”, “career OR work”, and “choice OR preference OR attitude”. Nine studies were eligible for inclusion. Nursing education may play a role in influencing how students perceive and prefer to work with older adults. Promising interventions include stand-alone gerontology courses, intergenerational service-learning experiences, and clinical experiences with community dwelling older adults. More evidence-based research with larger sample sizes are needed to determine effective nursing education interventions to improve nursing students’ attitude and preference for working with older adults.

